# Prevalence of SARS-CoV-2 Antibody in Healthcare Workers in Central Pennsylvania

**DOI:** 10.1017/ash.2021.93

**Published:** 2021-07-29

**Authors:** Taesung Kwon, Stacy Kenyon, Kimberly Kilheeney, Stanley Martin, Mark Shelly

## Abstract

**Background:** Determining the incidence of SARS-CoV-2 in healthcare workers (HCW) is important in assessing the safety of the work environment. Though of limited use in acute illness, serologic testing can detect some infections that occur undetected. We compared the prevalence of antibodies to SARS-CoV-2 to a place of work, exposure by role and department, and use of various prevention methods. **Methods:** Healthcare workers (HCWs) working in Geisinger Health System were offered voluntary serology through Employee Health. Before they had blood taken, they completed a brief questionnaire. Testing was conducted from June 15 to September 4, 2020. Blood was analyzed for SARS-CoV-2 immunoglobulin G (IgG) (Roche and Diasorin platforms). **Results:** In total, 2,295 employees and contract workers providing care at Geisinger facilities were tested. Most of this group, 2,037 (88.8%), were involved in direct patient care. In total, 101 tests returned positive, a rate of 4.4% (95% CI, 3.6%–5.3%). Of 54 HCWs with a positive NAAT for SARS-CoV-2, positive serology results were found in 48, a sensitivity of 89% (95% CI, 78%–95%). Those involved in patient care were slightly more likely to become infected, 91 of 2,037 (4.6%) compared to 10 of 258 who were not involved in patient care (3.9%; *P* = .68). Those with unprotected exposure to a known case of COVID-19 were more likely than those not exposed to be positive for SARS-CoV-2, 51 of 792 (6.4% vs 3.3%; *P* = .0008). This risk was highest for those exposed outside work (7 of 33; 21%; *P* = .003). HCWs working in COVID-19 units were positive at a rate of 4.0% (95% CI, 3.8%–5.4%), no more than other inpatient areas, which were 5.0% positive (95% CI, 3.8%–6.4%). HCWs working with outpatients were at slightly lower risk, 2.8% positivity (95% CI, 1.9%–4.1%). The rates of infection ranged between 3.3% and 5.0% by job category. Employees were asked about symptoms experienced since March 2020. Positive serology occurred in 39 (2.8%) of 1,414 employees who did not recall any symptoms. Symptoms related to COVID-19, except sore throat, were strongly correlated with positive serology. **Conclusions:** When provided a safe work environment, the risk of COVID-19 in employees is comparable to that in the surrounding communities. Persons with patient care responsibilities have an absolute risk that is marginally higher.

**Funding:** No

**Disclosures:** None

